# An updated meta-analysis of the distribution and prevalence of *Borrelia burgdorferi* s.l. in ticks in Europe

**DOI:** 10.1186/s12942-018-0163-7

**Published:** 2018-12-04

**Authors:** Agustín Estrada-Peña, Sally Cutler, Aleksandar Potkonjak, Muriel Vassier-Tussaut, Wim Van Bortel, Hervé Zeller, Natalia Fernández-Ruiz, Andrei Daniel Mihalca

**Affiliations:** 1 Department of Animal Health, Faculty of Veterinary Medicine, Miguel Servet 177, 50013 Zaragoza, Spain; 2 School of Health, Sport and Bioscience, University of East London, London, UK; 3 Department of Veterinary Medicine, Faculty of Agriculture, University of Novi Sad, Novi Sad, Serbia; 4 INRA, UMR BIPAR INRA, ENVA, Anses, Maisons-Alfort, France; 5 Surveillance and Response Support Unit, Solna, Sweden; 6 Present Address: Institute of Tropical Medicine, Unite of Medical Entomology, Antwerp, Belgium; 7 Office of the Chief Scientist Unit, European Centre for Disease Prevention and Control, Solna, Sweden; 8 Department of Parasitology and Parasitic Diseases, University of Agricultural Sciences and Veterinary Medicine Cluj-Napoca, Cluj-Napoca, Romania

**Keywords:** *Borrelia burgdorferi* s.l., Meta-analysis, Distribution, Europe, Modelling of prevalence

## Abstract

**Background:**

The bacteria of the group *Borrelia burgdorferi* s.l. are the etiological agents of Lyme borreliosis in humans, transmitted by bites of ticks. Improvement of control measures requires a solid framework of the environmental traits driving its prevalence in ticks.

**Methods:**

We updated a previous meta-analysis of the reported prevalence of *Borrelia burgdorferi* s.l. in questing nymphs of *Ixodes ricinus* with a literature search from January 2010–June 2017. This resulted in 195 new papers providing the prevalence of Bb for 926 geo-referenced records. Previously obtained data (878 records, years 2000–2010) were appended for modelling. The complete dataset contains data from 82,004 questing nymphs, resulting in 558 records of *B. afzelii*, 404 of *B. burgdorferi* s.s. (only 80 after the year 2010), 552 of *B. garinii*, 78 of *B. lusitaniae*, 61 of *B. spielmanii*, and 373 of *B. valaisiana*. We associated the records with explicit coordinates to environmental conditions and to a categorical definition of European landscapes (LANMAP2) looking for a precise definition of the environmental niche of the most reported species of the pathogen, using models based on different classification methods.

**Results:**

The most commonly reported species are *B. afzelii*, *B. garinii* and *B. valaisiana* largely overlapping across Europe. Prevalence in ticks is associated with portions of the environmental niche. Highest prevalence occurs in areas of 280°–290° (Kelvin) of mean annual temperature experiencing a small amplitude, steady spring slope, together with high mean values and a moderate spring rise of vegetation vigor. Low prevalence occurs in sites with low and a noteworthy annual amplitude of temperature and the Normalized Difference Vegetation Index (colder areas with abrupt annual changes of vegetation). Models based on support vector machines provided a correct classification rate of the habitat and prevalence of 89.5%. These results confirm the association of prevalence of the three most commonly reported species of *B. burgdorferi* s.l. in Europe to parts of the environmental niche and provide a statistically tractable framework for analyzing trends under scenarios of climate change.

**Electronic supplementary material:**

The online version of this article (10.1186/s12942-018-0163-7) contains supplementary material, which is available to authorized users.

## Background

The genus *Borrelia* is a large assemblage of bacterial species, which gained a great deal of attention in the last decades due to their importance for human health [1–3]. Lyme borreliosis, caused in humans by several species in the *Borrelia burgdorferi* s.l. complex (hereinafter Bb), is considered the archetype of an emerging infectious disease [4]. Information on the geographic distribution of the different species of Bb is of great epidemiological importance because the different species are associated with different clinical outcomes. Recent studies of the distribution of Bb in Europe focused on geographic analyses, based on administrative divisions, which do not follow natural ecological regions and cannot address the importance of environmental traits in the reported prevalence of Bb in ticks [5]. These bacteria circulate in natural complex cycles involving ticks and vertebrates. The large complexity of the natural networks allowing circulation of Bb has already been pointed out [6]. Some “bridge ticks” [7] act as transmitters of the pathogen to humans, while other tick species might support circulation of Bb in silent foci. These ticks do not commonly bite humans, therefore the presence of the pathogen is hard to realize in the absence of human clinical cases.

The spatial distribution of the medically relevant tick vectors in Europe (*I. ricinus* and *I. persulcatus*) is far from uniform. It has been suggested that the adaptations of tick species and vertebrate hosts to the local climate contribute to the spatial distribution patterns of Bb [8–10]. It is well known that Bb species circulate in different vertebrate reservoirs [9]. Further, a recent study suggested a clear association between genotypes of Bb species and tick species [6, 11]. It is thus necessary to consider the associations between ticks, reservoirs and environment to understand the occurrence of Bb. Hence, changes in the distribution of both the vector and the reservoirs is expected to impact the spatial distribution of Bb in Europe [12].

The prevalence and distribution of Bb in *I. ricinus* in Europe have been reviewed [13, 14] and meta-analyses of the prevalence of Bb species in *I. ricinus* ticks in Europe have already been performed [5, 15, 16]. The distribution of *I. ricinus* and *I. persulcatus* continues to expand northwards in latitude and upwards in altitude in Europe [12, 17–24]. Climate trends and the density of key hosts for the adults of the tick, have been pointed as the main factors behind the spread of *I. ricinus* [12, 23, 25].

Considering all these aspects, it is therefore necessary to (1) update previous meta-analyses of the distribution of Bb ticks in Western Palearctic, (2) improve our knowledge of the determinants of the observed distribution of Bb in ticks, and (3) build a synthetic explanation of such interacting factors driving the reported prevalence in questing ticks. Here, we updated our previous meta-analysis [16] of the reported prevalence and distribution of Bb in host-seeking *I. ricinus* ticks in Europe based on a systematic literature review. We also included in our analysis *B. miyamotoi*, a relapsing fever species, because of the growing concerns about its importance [26]. In this study we (1) describe the occurrence and prevalence of the species of Bb in *I. ricinus* in Europe and (2) the environmental traits that drive the reported prevalence of the most common Bb species in questing *I. ricinus*.

## Materials and methods

### Data collection, assemblage of the primary dataset and data cleaning

**Fig. 1:**
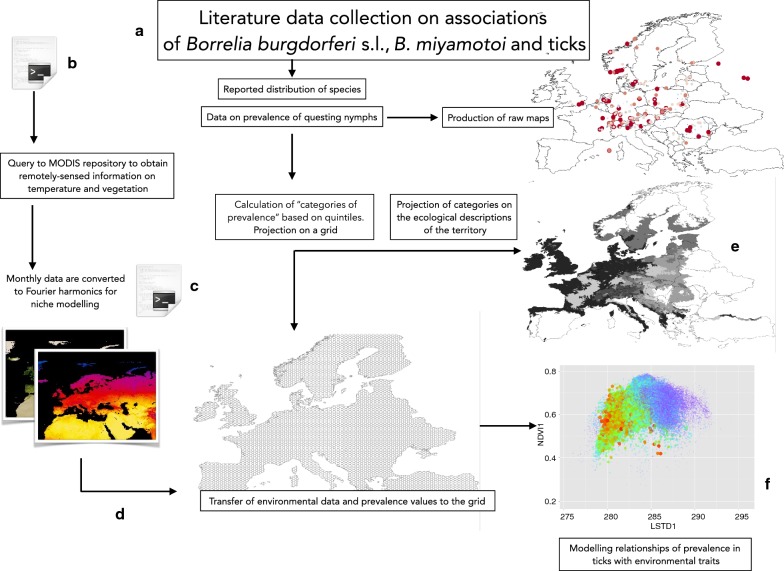
The workflow of the study. We collected literature data (**a**) in the period 2010–2017 about the prevalence of *Borrelia burgdorferi* s.l. (Bb) and *B. miyamotoi* in questing ticks (but only data from questing nymphs were finally used). This allowed to plot raw maps of the reported distribution of each species of Bb. We built scripts in R to mass download 8-days 1 km. satellite images from MODIS repository (**b**) that were transformed into Fourier components using in-house developed scripts (**c**). All the scripts and the transformed satellite imagery are available in http://datadryad.org/resource/doi:10.5061/dryad.2h3f2.2. We tessellated the complete territory with a grid of hexagonal cells of 0.25° of radius, and environmental data as well as prevalence values (converted to categories) were transferred (**d**). The categories of prevalence for each species were also transferred (**e**) to a standard definition of the ecological classes in Europe (LANMAP2) to obtain wide area estimates of Bb species composition and prevalence. Data on prevalence and environmental traits were used to produce a map in the environmental niche about the restriction of Bb species and prevalence to different portions of the niche outlined by temperature and vegetation (**f**). These grid-derived values were also used to model the influence of the environmental traits on the observed values of prevalence by training a Neural Network

This study is based on the collection of published reports about the prevalence of Bb in questing nymph ticks (see Fig. 1 for data collection strategy). We also included data on *B. miyamotoi.* We did not include the prevalence of Bb in wild, domestic animals or humans, nor data reported in feeding ticks. The former tends to introduce bias in the reports, because some vertebrates may be more efficiently trapped compared to scarce or protected species, which are therefore under-represented in reports even if they prominently harbour Bb. The latter always distort the rates of carried micro-organisms in ticks, because DNA amplification methods cannot differentiate between DNA of the pathogen that was already in the tick or was a consequence of the blood meal [27].

The bibliographical search was performed in PubMed, Google Scholar, and The Web of Knowledge, for the period January 2010–June 2017 using the words (“*Ixodes*” OR “Ixodidae” OR “Tick*”) AND (“*Borrelia*”) AND (“Europe” OR [list of countries of European Union, EEA, and Balkan countries]). This query was deliberately relaxed since we observed in preliminary tests that other combinations of keywords resulted in a loss of published papers reporting the prevalence of Bb in ticks. The target territory included the 28 European Union Member States together with Iceland, Liechtenstein, Norway, Switzerland and the Balkan countries. Title and abstract were screened to remove the papers not dealing with the prevalence of Bb in ticks. Papers describing mainly clinical manifestation(s) in humans or prevalence of Bb in vertebrates were included in the dataset only if they reported also data of prevalence in questing ticks. Papers reporting the results as “*B. burgdorferi* s.l.” were used for updating the main distribution data, even if no names of species were provided, but data were not used for modelling. We included only reports with a pair of coordinates or for which the name of the collection locality could be unambiguously referred to a pair of coordinates. The final dataset of the period 2010–2017 was updated with geo-referenced data of prevalence of Bb in questing ticks from a previous, similar study [16]. Only data from questing nymphs were used, since adult ticks are more infrequently collected, and they had two different blood meals, thus complicating further analyses. Moreover, nymphs are the most common stage feeding on humans. Some papers reported the data referred to pools, and subsequently the prevalence in each pool was used.

A critical issue in this kind of meta-analysis is the number of ticks collected, analyzed and reported. To avoid bias in the modelling of the data, we included only studies that comprised a sufficient number of nymphal ticks in the study. We established the threshold of the minimum sample size as the 10th percentile of the dataset. This corresponds to a minimum of 29 questing nymphs for each sampling point. This choice was done after a visual inspection of the number of ticks tested against the prevalence: lower numbers of ticks were always associated with higher prevalence, therefore biasing the outcome of the analysis. We did not test the potential influence of a lower number of ticks on the modelling: we thus adhered to the inclusion of a minimum number of ticks by report based on the pure frequency distribution of the data. The phenology of the tick and the date of collection were not considered, and we refer to the “site” (i.e. the pair of coordinates) independently of the number of surveys carried out in a period of time in the same site, including only those which were above the minimum threshold, and averaging the values of prevalence.

## Sources of climate and ecological data

We aimed to establish a link between the environmental niche and prevalence of Bb in questing nymphal *I. ricinus* according to a scheme with ecological meaning. We exploited a series of remotely sensed variables describing the ground temperature (in Kelvin) and the Normalized Difference Vegetation Index (NDVI). The NDVI is a measure of the photosynthetic activity of the vegetation, and is influenced by the types of canopy and soil and the leaf shape. However, it is known that the length of the vegetative period (as recorded by NDVI) is an important feature for capturing the distribution of ticks and the presence/absence of Bb [21]. For modelling purposes, we used the Fourier-transformed remotely sensed imagery because it represents the best balance between a low number of explanatory variables with an adequate description of the climate traits [6, 28]. The Fourier transform (or harmonic regression) uses a series of weather data to extract its main factors, as the mean of the series of values, the slope at the spring or the (negative) slope of autumn. We demonstrated that only three variables account for the mean values of temperature, the spring rise, the duration of the summer, the decrease and the negative slope in autumn, or the duration and mean values of temperature of autumn and winter, which adequately represent the environmental niche of ticks [28]. We already made publicly available the raster explanatory variables and the scripts to obtain them from time series data, which can be accessed at http://datadryad.org/resource/doi:10.5061/dryad.2h3f2.2. We used three variables for temperature and three for NDVI.

We used the GlobCover system to obtain the dominant vegetation and the fragmentation of the territory, to be used together with the climate variables as ecological descriptors of the distribution and prevalence of Bb. GlobCover is a global classification based on ENVISAT’s MERIS satellite, at a resolution of 300 meters (available at http://geodata.grid.unep.ch/mod_download/download.php, accessed August, 2017). Other European standards, like CORINE, lack the adequate number of categories necessary to describe the habitat of ticks.

## Analysis of the data and statistical methods

### Plotting the prevalence of Bb in questing nymphs

We did crude plots of the reported prevalence of every species of Bb. We did not attempt to extrapolate any conclusion about trends of prevalence over time, using data for the periods 2000–2010 and 2010–2017. The patchy nature of the reports, randomly covering small portions of the wide target territory (i.e. surveying rather small territories for periods of different time length) precluded an assessment of trends over time for prevalence of Bb in questing nymphs.

### General procedures

We first tessellated the target territory with a grid of hexagons, 0.25° (decimal degrees) radius for each cell. Both dominant vegetation and the number of different classes of vegetation were obtained for each cell of the grid covering the target territory. We included in each cell of the grid the climate-derived explanatory variables. Data on dominant vegetation (the most represented category of vegetation) and habitat fragmentation (the number of different vegetation categories) in each cell of the grid were added to the satellite-derived weather variables to explain the prevalence of Bb in questing ticks. Other than the tessellation of the territory for modelling purposes, we aimed to link the prevalence of Bb across rough descriptions of the climate of Europe. We adhered to previous approaches [29, 30] using the European Landscape Map, LANMAP2, a pan-European landscape database at a scale of 1:2,000,000 [31]. LANMAP2 is a hierarchical classification with four levels and has 350 landscape types at its lowest level. At this level there are more than 14,000 mapping units with an average size of 774 km^2^. The highest level of the classification is determined by climate and has only eight classes; the smallest mapping unit is 11 km^2^. These raw data are available as Additional file 1, and include the original records with coordinates, as well as the environmental variables associated with each record, including continuous (i.e. MODIS-derived) and categorical data (i.e. LANMPA2 and GlobCover) as used for further statistical processing.

### Capturing the relationships between the environmental niche and the prevalence of Bb in questing ticks

We used the grid data to model the relationships between combinations of climate and landscape variables with the prevalence of Bb in questing nymphs of *I. ricinus*. The cells of the grid covering the territory for which prevalence data exist were loaded with the reported value(s) of prevalence, as the variable to be explained by the climate and landscape (dominant vegetation and habitat fragmentation) traits. The rather disparate prevalence of Bb in questing nymphs in geographically close surveys produced a background noise, since two or more reports with very different prevalence values could overlap in the same cell. In this case, if two or more reports were in the same cell of the grid, the weighted average of the reported prevalence, based on the number of processed ticks, was used.

For the same reasons, we opted to reclassify the values of prevalence of Bb in the cells of the grid into categories. We used the quintiles of the frequency distribution of the reported prevalence of each Bb species in questing nymphs to establish the following categories: “absent” (< 1%), “very low” (1–4%), “low” (4–9%), “medium” (9–21%), “high” (21–46%) and “very high” (> 46%). Some species, like *B. spielmanii*, *B. finlandensis*, or *B. miyamotoi* (not a Bb species) were scarcely reported (less than 10 reports) or even not reported in questing nymphs of *I. ricinus* and were therefore not subjected to further statistical analyses. Table 1 includes the number of records available for the categories of prevalence of *Borrelia afzelii*, *B. garinii* and *B. valaisiana*, which were the most commonly reported species of Bb.

**Table 1:** The categories of prevalence in questing ticks of the most frequently reported species of *Borrelia* spp. in the target territory

Category	*Borrelia afzelii*	*Borrelia garinii*	*Borrelia valaisiana*
Absent	42	55	65
Very low	106	143	116
Low	131	123	91
Medium	132	99	70
High	66	74	21
Very high	81	58	10
Total	558	552	373

Categories are “absent” (< 1%), “very low” (1–4%), “low” (4–9%), “medium” (9–21%), “high” (21–46%) and “very high” (> 46%)

We trained models built with Neural Networks, Naive Bayes, AdaBoost (short for Adaptive Boosting) and support vector machines in the “Orange” programming environment version 3 (https://orange.biolab.si) aimed for developing the best model classifying each cell according to the class of prevalence of Bb. The selection of algorithms was not intended to be exhaustive, and we used the four methods producing the best results in preliminary tests. For every method, models were trained with a random selection of 50% of grid cells of each category. However, algorithms have different sensitivity to data imbalance. The only condition for data entering was that the total number of cells in the category “absent” was not higher than the sum of the grid cells of the other categories. The “absent” category can produce unexpected results because the “absence of Bb” could be derived from its real absence or because the randomly chosen grid entering into the training set has not been surveyed. To avoid this potential bias, we included the condition of “vector being present” as an additional condition; therefore, only the grid cells with the category “Bb absent” and vector “present” were introduced in the training set for modelling.

The best neural network was obtained with 100 neurons in one hidden layer, activated by the ReLu (rectified linear unit) function and the L-BFGS-B solver, with alpha = 0.0001 and 200 maximum iterations. For Naive Bayes we assumed equiprobable classes (i.e., priors = 1/(number of classes)). For AdaBoost, we used the option “tree” as base estimator, with 50 estimators and a learning rate of 1, using the SAMME.R as classification algorithm and the linear regression loss function. For SVM, we used a RBF kernel, with a maximum of 100 iterations and a tolerance 0.001. These values were selected after preliminary tests aimed to choose the best combination of base estimators. The data on the effects of parameter selection in modelling are not explicitly included but are available using Additional files 2 and 3. Because of limited data availability for other species of Bb, we performed the modelling only with the best represented ones, namely *B. afzelii*, *B. garinii* and *B. valaisiana*. The script for modelling based on the “Orange” programming environment is available as Additional file 2, together with the dataset entered to produce the models, available as Additional file 3. The script needs the download of the software (freely available) to run.

## Results

### Description of the reported data

We compiled a total of 195 papers providing the prevalence of Bb in questing *I. ricinus* in the period January 2010–June 2017. The reports of this period included a total of 36,894 ticks. The complete dataset of prevalence in questing nymphs, with adequate identification of the Bb species and with a sample size of nymphs above the 10% of the frequency distribution consisted in 1177 records, of which 926 had adequate information about coordinates. A further set of 878 records (years 2000–2010, with geo-references, see Estrada-Peña et al. [16]) has been added to the 2010–2017 dataset. This produced a total of 558 geo-referenced records of *B. afzelii*, 404 of *B. burgdorferi* s.s. (only 80 after the year 2010), 552 of *B. garinii*, 78 of *B. lusitaniae*, 61 of *B. spielmanii*, 373 of *B. valaisiana*, and 29 of *B. miyamotoi*. *Borrelia finlandensis* and *B. turdi* have been reported (2010–2017) only in feeding ticks and/or in host tissues, and these records are therefore not included.

**Fig. 2:**
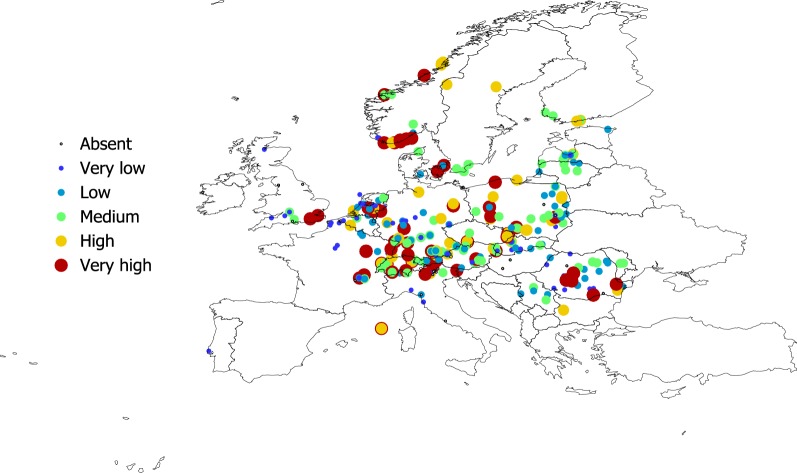
The reported distribution and prevalence of *B. afzelii* in questing nymphs of *I. ricinus* (data for years 2010–2017). The size and the levels of grey of the plots in the legend define the prevalence at the coordinates of the points

**Fig. 3:**
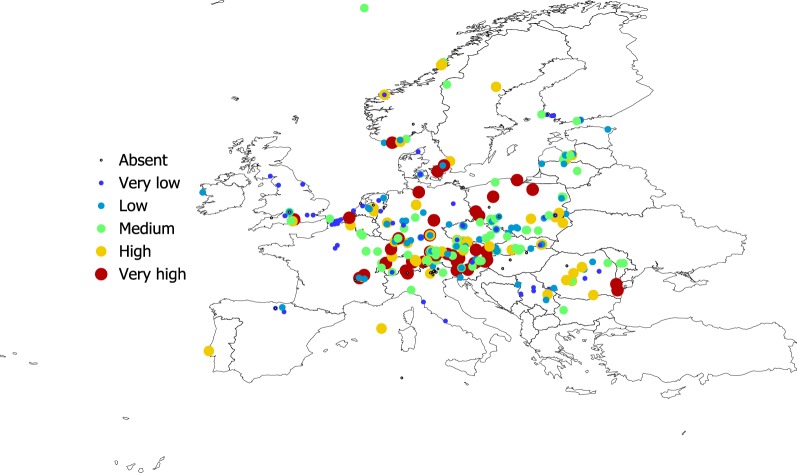
The reported distribution and prevalence of *B. garinii* in questing nymphs of *I. ricinus* (data for years 2010–2017). The size and the levels of grey of the plots in the legend define the prevalence at the coordinates of the points

**Fig. 4:**
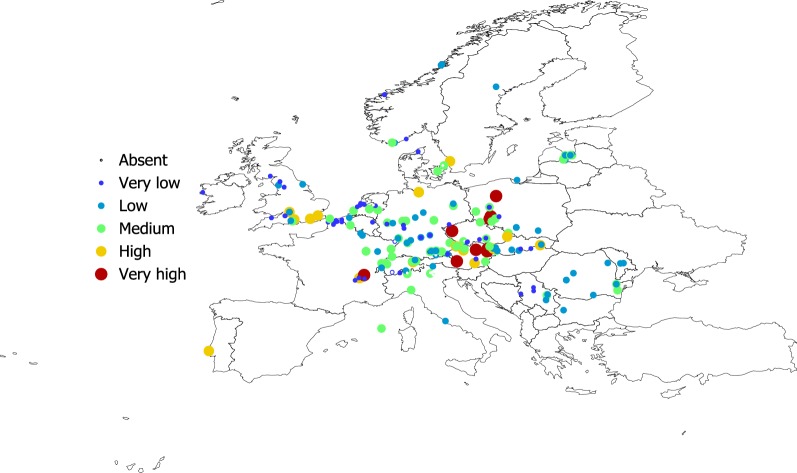
The reported distribution and prevalence of *B. valaisiana* in questing nymphs of *I. ricinus* (data for years 2010–2017). The size and the levels of grey of the plots in the legend define the prevalence at the coordinates of the points

**Fig. 5:**
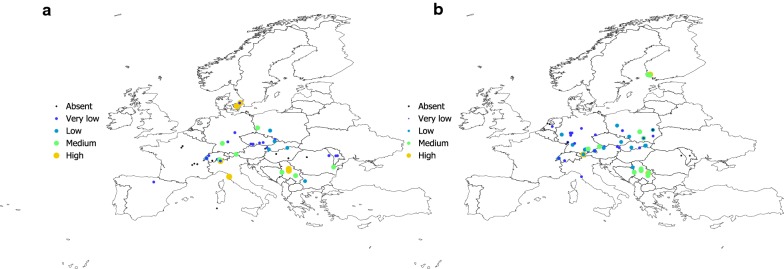
**a** The reported distribution and prevalence of *B. lusitaniae* in questing nymphs of *I. ricinus* (data for years 2010–2017). The size and the levels of grey of the plots in the legend define the prevalence at the coordinates of the points. **b** The reported distribution and prevalence of *B. burgdorferi* s.s. in questing nymphs of *I. ricinus* (data for years 2010–2017)

**Fig. 6:**
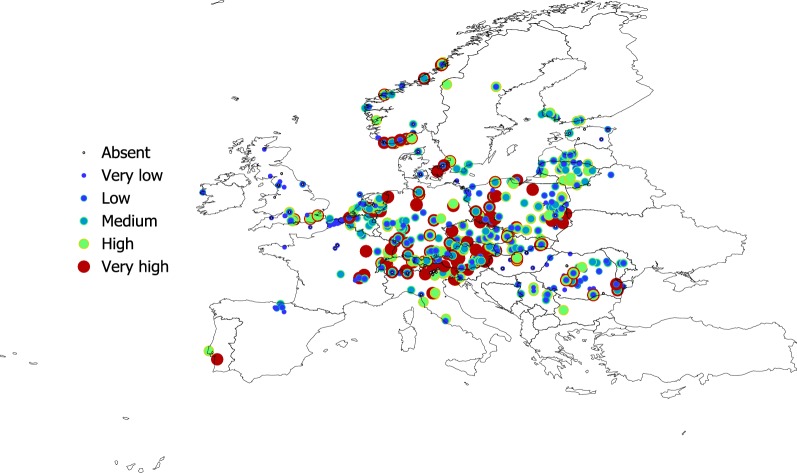
The reported distribution and prevalence of *B. burgdorferi* s.l. in questing nymphs of *I. ricinus* (data for years 2000–2017). The size and the levels of grey of the plots in the legend define the prevalence at the coordinates of the points

Figures 2, 3, 4 and 5 show the geographical pattern of prevalence of the five species of Bb in questing nymphs for which examined specimens were above the threshold limit, over the period 2000–2017. Figure 6 displays the overview of the prevalence of Bb s.l. in questing nymphs in the target territory. The figure has been produced with reports resulting from 82,004 questing nymphs (years 2000–2017), after the removal of the records reporting a sample size below the 10% threshold established for analysis. The visual inspection of these figures does not provide clues about a spatial pattern for the species of Bb or their prevalence in questing nymphal ticks. The main point from these maps is that Bb exists at very variable rates anywhere the vector exists, with maximum prevalence at large regions of central Europe. The pathogen tends to be less prevalent at the fringe of its distribution, probably because the colder conditions restricting vector resilience (i.e. Baltic countries) or because the lack of adequate reservoirs (i.e. United Kingdom). In any case, the reported prevalence is very patchy across the target territory. It is necessary to observe that the patchy spatial pattern of presence-absence is the consequence of a meta-analysis, in which the surveys have not been previously planned. However, the sudden changes of prevalence for spatially near reports should be considered as the actual patchy distribution of these rates.

**Fig. 7:**
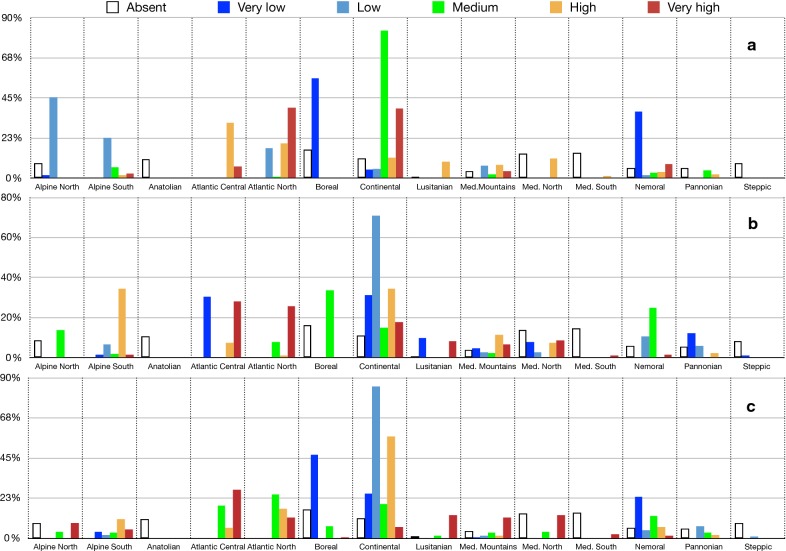
The percentage of prevalence classes of *B. burgdorferi* s.l. in questing nymphs of *I. ricinus* associated with the climate zones in the target region (data for years 2000–2017). The X (categorical) axis shows the types of climate according to the definition of LANMPA2. The Y axis shows the percent of the prevalence categories as distributed across the climate categories

The cross-tabulation histogram of the prevalence of *B. afzelii*, *B. garinii*, and *B. valaisiana* against the general types of climate (as provided by LANMAP2 classification) is included in Fig. 7. This cross-tabulation produced observable differences between the prevalence of *B. afzelii* and the other species. The former seems to be more common in regions with a Continental climate, while both *B. garinii* and *B. valaisiana* are more prevalent in regions of Mediterranean mountains or Lusitanian climate. Some categories of prevalence have been more commonly reported in Central and North Atlantic regions (high prevalence of *B. afzelii* together with different combinations of *B. garinii* and *B. valaisiana*), Continental and Boreal-Nemoral classes of climate.

### Modelling uncovers a pattern of spatial distribution for Bb in Europe

**Fig. 8:**
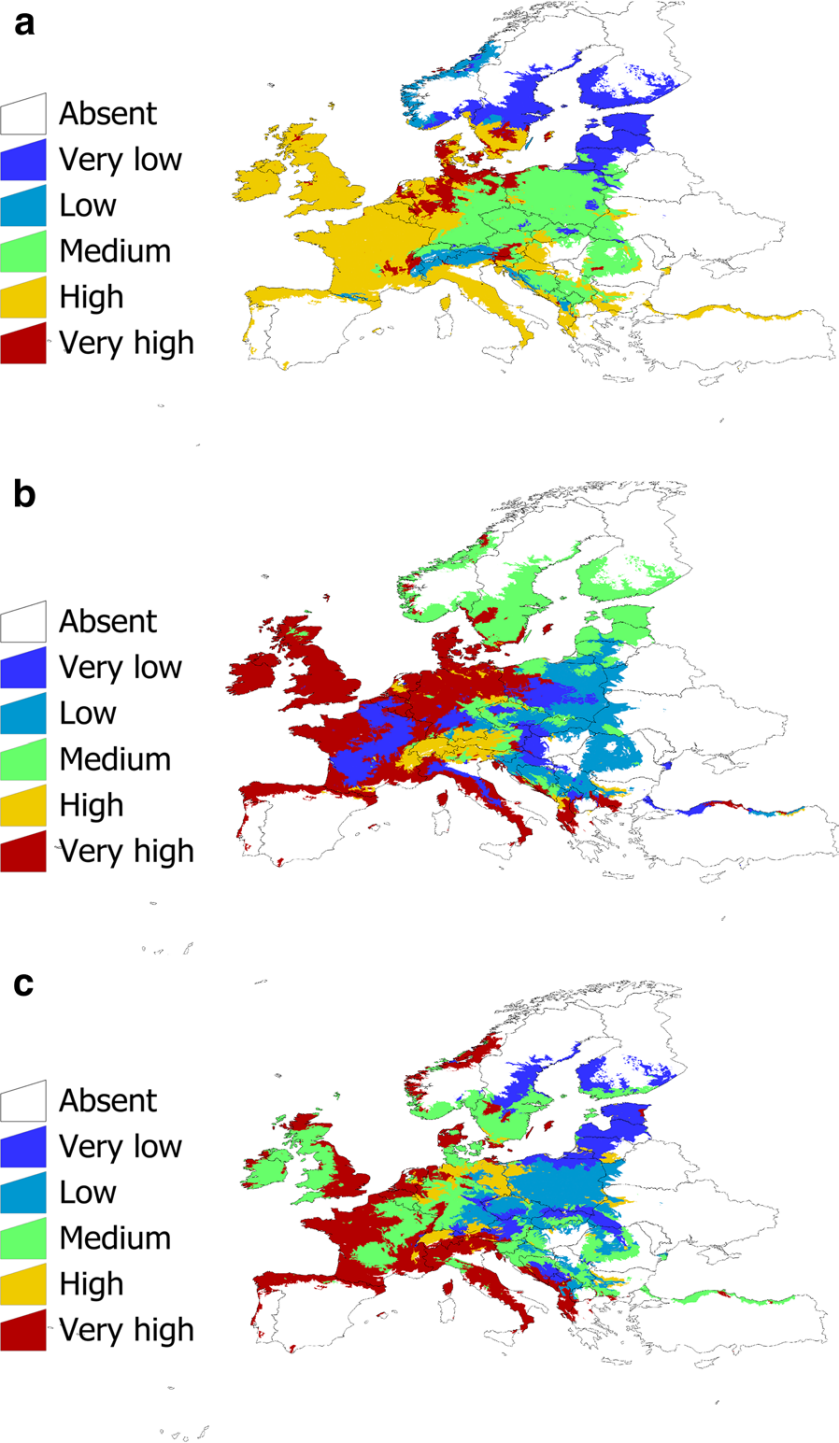
The projection of the prevalence categories of *B. afzelii* (**a**), *B. garinii* (**b**), and *B. valaisiana* (**c**) modelled using support vector machine on the climate categories (LANMAP2 scheme) of the target territory

The classification of the categories of prevalence produced good modelling results, using only temperature and NDVI -derived variables. The SVM over-performed the rest of the modelling approaches (Table 1). The SVM produced a correct classification rate of more than 98% and 99% (the former for *B. afzelii* and *B. garinii*, the later for *B. valaisiana*), with an AUC (area under the curve, commonly used to explain together true positives and false positive rates) higher than 0.81 for every species (in the range 0–1). We thus used SVM, the six temperatures and NDVI variables, to classify the target territory with the predicted prevalence of these three species, and the results were plotted over the LANMAP2 bioclimate regions of the target territory (Fig. 8). It resulted that the prevalence categories of the three most reported species of Bb shows a geographical pattern, unforeseeable from the representation of the crude prevalence as reported. While the three species show a wide distribution in Europe, the expected prevalence of each species is very different. *Borrelia afzelii* tends to be common in most of Central Europe and Atlantic regions, with low prevalence in the periphery of its distribution. The prevalence of *B. garinii*, however, shows a patchier distribution, with large areas of the Atlantic and central European regions included in the “very high” category. *Borrelia valaisiana* is also expected to be frequent in questing nymphal *I. ricinus* mainly in coastal areas and central Europe, with medium values of prevalence in central Europe. All these three species tend to be rare in the margins of their range (Table 2).

**Table 2:** Performance of the four classification methods used to predict the prevalence of *B. afzelii*, *B. garinii*, and *B. valaisiana* in a grid of 0.25° radius over Europe using a combination of environmental data

	*B. afzelii*		*B. garinii*		*B. valaisiana*	
	AUC	CA	AUC	CA	AUC	CA
Neural Networks	0.491	0.972	0.585	0.963	0.496	0.989
Naive Bayes	0.550	0.626	0.592	0.662	0.824	0.775
AdaBoost	0.499	0.974	0.500	0.961	0.500	0.989
SVM	0.816	0.987	0.816	0.981	0.844	0.995

*AUC* area under the curve, *CA* classification accuracy. The AUC values refer to the average of all the classifications. Complete AUC and CA values for each category can be obtained from the Additional files 2 and 3

### The prevalence of Bb is associated with environmental features

**Fig. 9:**
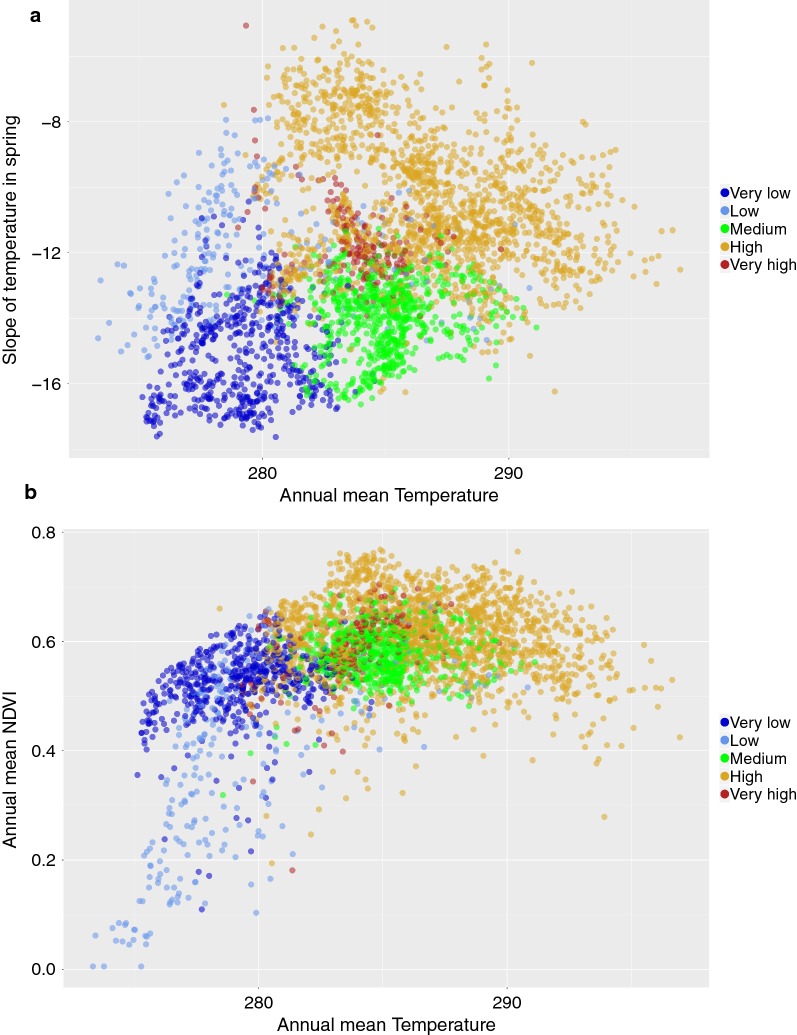
The distribution of major classes of prevalence of *B. afzelii* in the environmental axes of **a** mean annual values of temperature (in Kelvin) *versus* the slope of temperature in spring (unitless), and **b** mean annual values of temperature *versus* the slope of NDVI in spring for the period 2002–2016. Points of the category “absent” were not plotted to improve readability

**Fig. 10:**
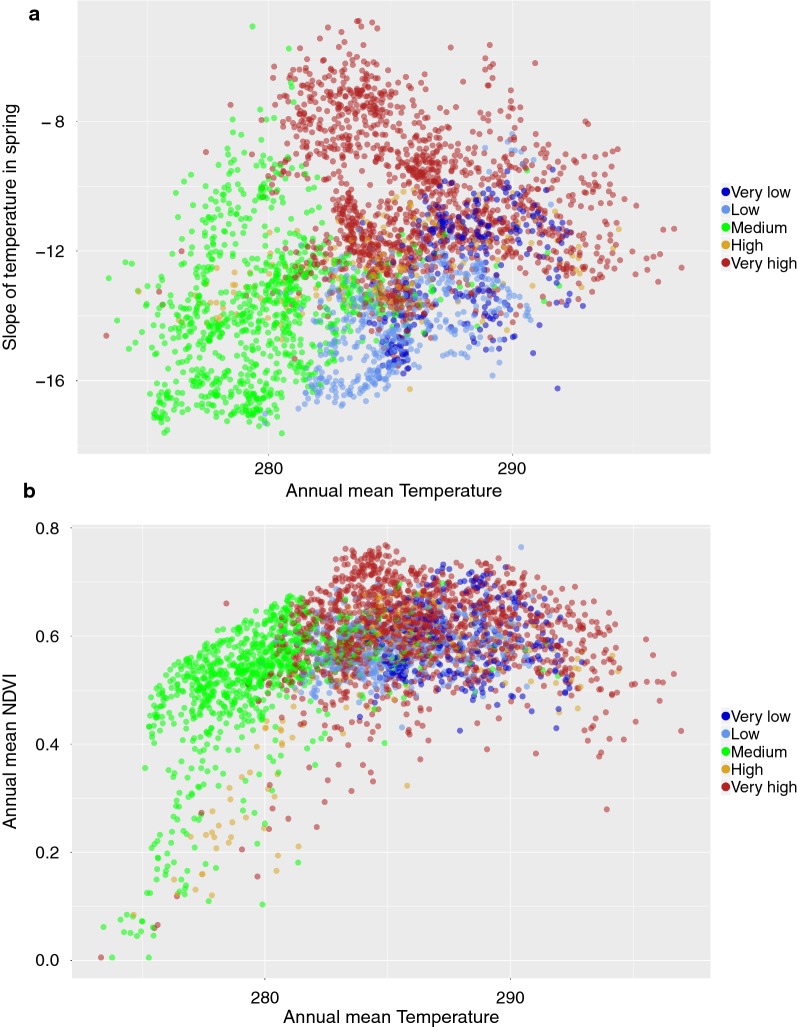
The distribution of major classes of prevalence of *B. garinii* in the environmental axes of **a** mean annual values of temperature (in Kelvin) versus the slope of temperature in spring (unitless), and **b** mean annual values of temperature *versus* the slope of NDVI in spring for the period 2002–2016. Points of the category “absent” were not plotted to improve readability

**Fig. 11:**
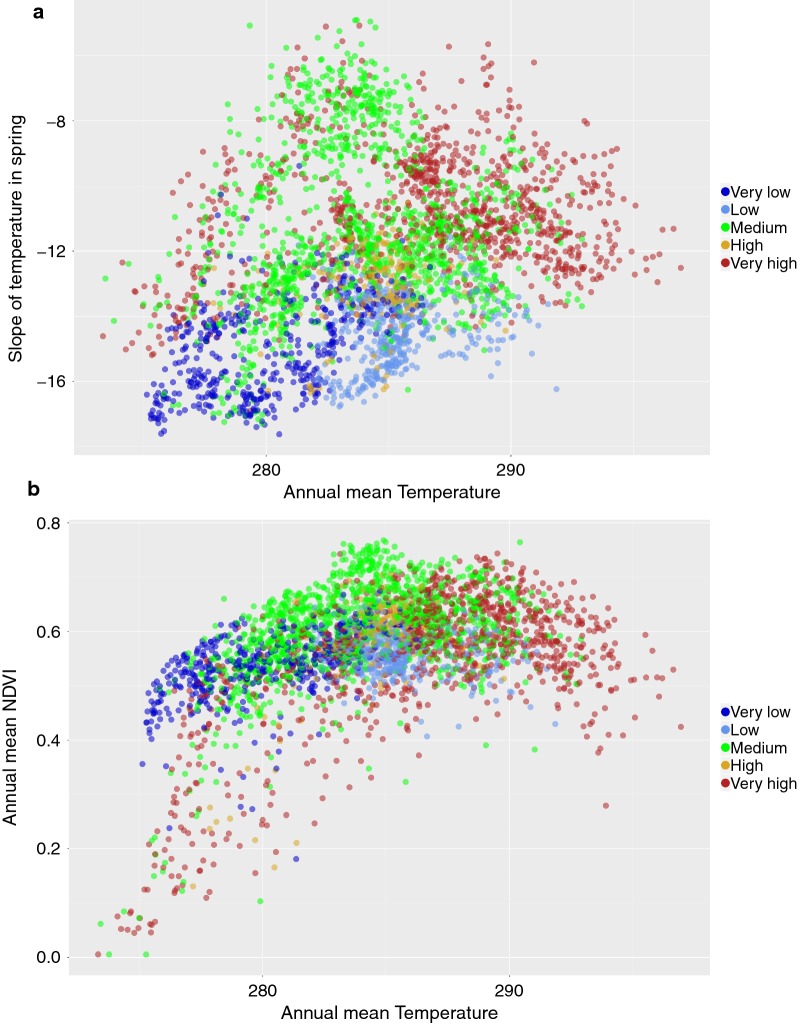
The distribution of major classes of prevalence of *B. valaisiana* in the environmental axes of **a** mean annual values of temperature (in Kelvin) *versus* the slope of temperature in spring (unitless), and **b** mean annual values of temperature *versus* the slope of NDVI in spring for the period 2002–2016. Points of the category “absent” were not plotted to improve readability

We aimed to visually capture the relationships of the predicted prevalence categories with the environmental traits for the three modelled Bb species. The focus was to check if there were “portions” of the environmental niche in which prevalence of categories were different, or, in other words, if the predicted prevalence is associated to a delimited portion of the environmental variables. This would result in a framework that could be subjected to analysis of change or resilience according to different climate scenarios. Figures 9, 10 and 11 group the plots of prevalence classes against two variables of temperature and one of NDVI (annual mean and slope in spring for temperature; annual mean temperature and annual mean NDVI). There is an overlap in some portions of the environmental niche defined by these variables: there is no evidence of an “environmental barrier” clearly separating prevalence classes. Most importantly, however, these three species of Bb co-occur at some portions of the environmental niche at different prevalence, delimited by the annual mean values and the slope in spring of both temperature and NDVI. The conclusion is that there are features in the environmental niche that promote different rates of co-occurrence of these species, revealed as different prevalence categories in close portions of the environment. Both *B. afzelii* and *B. garinii* tend to occur at different parts of the niche defined by the temperature, but not well delimited by NDVI. *Borrelia valaisiana* shows a patchier distribution along the main axes of the environmental niche (something already observed in the geographic space). With the obvious hesitation regarding the modelling procedures and the unplanned surveys resulting in a meta-analysis, these results indicate that environmental variables are associated to the prevalence categories of the three most reported species of Bb in Europe.

## Discussion

This study updates a previous meta-analysis [16] on the prevalence of Bb in questing nymphs of *I. ricinus* in Europe using reports published between 2010 and mid-2017, improving our knowledge of the range of Bb in Europe. The data for the period 2010–2017 (> 36,000 questing nymphs) were updated with data from the period 2000–2009 (> 43,000 questing nymphs). The current study thus represents the largest analysis of the distribution of the pathogen in questing nymphs of *I. ricinus* in Europe. We used only data from questing nymphs because of the unreliability of reports in ticks collected and processed while feeding; questing nymphs are also the more abundantly collected stage of *I. ricinus*. This study has obvious limitations that emanate from the structure of the data sources. We could not analyze temporal trends from the reports because of the lack of a coherent background, which should be ideally structured around the same methods. While we acknowledge that a temporal analysis of prevalence trends for Bb in questing ticks would be necessary, meta-analysis is not an adequate framework if surveys have used heterogeneous methodological approaches. A meta-analysis is built from unplanned surveys, with data obtained using a large variety of methods with different reliability. Also, the spatial resolution used in this study, aimed to provide a coherent background at a relatively rough resolution, cannot capture the local nature of the foci of Bb. Moreover, the systematics of this group of bacteria have undergone recent important changes [9]. Consequently, caution is needed when examining old reports of some species. We prefer to be conservative about these data. For example, most records of *B. burgdorferi* s.s. (2000–2010) were published before the description of new species within the group [32–34] with a sudden drop of records correlating with use of probes that are able to separate species of Bb. It is a well contrasted fact that *B. burgdorferi* s.s. tends to be rare in the Western Palearctic (Margos, pers. comm.). This is why *B. burgdorferi* s.s. was not included in modelling, because reliable records are scarce, and old records (approximately pre-2010) might actually correspond to other species.

Our results provided additional support to previous reports about the spatial prevalence of Bb in questing nymphs of *I. ricinus*: the surfaced pattern shows the highest prevalence and the largest combination of Bb species in parts of central Europe, associated with Continental or North Atlantic-type of climates. These findings contradict early reports [7, 15, 35] a fact probably derived from the bias produced by tabulating reports according to administrative divisions. In general, the updated results outline the already reported range of Bb [16]. However, the focus of the current study on the most frequently reported species and the use of categorical values of prevalence produced a sharper picture of both the spatial pattern of Bb and the relationships of prevalence with the environmental traits.

The raw quantitative values of prevalence produced a noise which was removed by partitioning the data using the quantiles of its distribution, producing a statistically coherent aggregation of data. Several other strategies have been employed in this study, to improve data mining, summarizing resulting information, and improving modelling, like (a) the use of satellite-derived climate data transformed after a harmonic regression, (b) summarizing of data over a tessellation of the territory, and (c) using methods for supervised classification of prevalence categories. However, the use of a limited number of classification methods could also result in a bias, since not every available method has been tested, and because some methods may perform better than others giving data imbalance and other peculiarities in the capture of the environmental niche associated with records of Bb [21]. We previously included in modelling the landscape fragmentation, which has been pointed as a driver of tick abundance [36], since habitat fragmentation promotes host movements [37]. Small patches would promote ecotones, which are interfaces between different types of vegetation, therefore allowing a greatest rate of hosts movements [13]. However, the modelling methods systematically rejected the inclusion of landscape patchiness in the best models, which agrees with the spatial scale used in this study for modelling (0.25°) probably too large to capture the intrinsic features of the habitats at a local scale. Collectively, the approach looks promising in evaluating the trends of a pathogen that is primarily driven by changes in climate the abundance of adequate reservoirs and social habits [38, 39].

We acknowledge that the structure of the data collection and the non-random reporting introduced a background noise, obscuring the set of ecological traits affecting the distribution of Bb [40–42]. However, the results confirm a macro-ecological pattern of niche availability for both the tick and the reservoirs, driving the prevalence of the most reported species of Bb. We believe that this effect may be primarily driven by the availability and relative abundance of the main reservoirs, as it has been reported [43]. However, these patterns of climate also delineate the phenology of both the ticks and the vertebrates, therefore shaping their contact rates not only in the geographical background but in the different moments of the year. All these features have a direct impact on the prevalence of Bb and could be behind the associations of the prevalence with defined portions of the environmental niche. It is of interest to point out that the most reported Bb species in Europe have the same spatial distribution, which clearly overlap with the known distribution of the tick *I. ricinus* [6, 44] but at different prevalence.

We thus propose that there is an environmental niche that delineates the prevalence of at least three species of Bb in ticks in Europe, which are otherwise widely distributed anywhere the vector exists. It also results that any analysis of the effects of climate on the contact rates between ticks and reservoirs should be measured in the environmental niche, not in the spatial range, as already proposed [44]. This turns the pure spatial distribution of the pathogen(s) into a statistically meaningful framework. It constitutes a valuable support to explore the impact of climate change on the occurrence of Bb in a large territory transformed into its niche defined by a combination of environmental variables, like temperature or NDVI. While such framework cannot address local processes, it could estimate long term trends in the prevalence and spread of a medically important bacterium, including future climate projections. It is important to “abandon the map” as the preferred worktable for these studies and assess the impact of change on the dimensions of the environmental niche. The maps of predicted prevalence in *I. ricinus* nymphs should be taken as an indication of exposure to Bb but not as a mirror of actual human incidence rates. This is widely acknowledged to occur [45] because the complex multi-strain, multi-host interactions associated with Bb infection make it difficult to determine the risk of infection to humans [46–48]. The lack of reliable data from many European countries, where the incidence in humans is not of mandatory reporting, further complicates the correlation of data between the prevalence of Bb in questing nymphs and its incidence in humans.

In conclusion, this study updated a previous meta-analysis of the occurrence rates of Bb in questing nymphs of *I. ricinus* in Europe. It confirmed previous findings on the largely overlapping distribution of several species of spirochetes reported in the target territory and demonstrated the widespread distribution of three main species. We demonstrated that continuous values of prevalence are of low utility for a solid statistical analysis, and that transformation of these rates into categories provide a better outlook on the epidemiological landscape. The modelling of the prevalence using satellite-derived climate and vegetation data, resulted in a low-resolution model predicting the occurrence of Bb. These results confirm that, at least for the three most commonly reported species of Bb, an association with parts of the environmental niche is evident, most notably involving not only mean values of temperature and NDVI but also the annual phenology of these variables.

## Conclusions

There is an environmental niche driving the prevalence of the most commonly reported species of bacteria of the *B. burgdorferi* s.l. complex in questing nymphs of *I. ricinus* ticks. Results obtained from a meta-analysis of more than 82,000 nymphs revealed that *B. afzelii*, *B. garinii* and *B. valaisiana* largely overlap across Europe. The highest prevalence occurs in areas of 280°–290° (Kelvin) of mean annual temperature (around central Europe and southern parts of Nordic countries) and a slow spring rise of temperature, together with high mean values and a moderate spring rise of vegetation vigor. Models using a support vector machine based on satellite derived, Fourier-transformed variables, identified ranges of prevalence for these three most commonly reported species in Europe. These results are of special interest not only for delineating areas of risk for humans, but also that the prevalence of the bacteria in ticks can be associated to models based on scenarios of climate change and used to track possible changes in prevalence.

## Additional files

**Additional file 1.** The complete raw data on the prevalence of *Borrelia* spp. in questing nymphs of *Ixodes ricinus*. The columns of the file include “Year Of study”, the “Species” of *Borrelia* spp., “Latitude” and “Longitude” of the original report, “Prevalence” of the original report, and “Sample” (number of questing ticks included in the original report). Included are also the values obtained from each environmental variable (TFourier1 to TFourier 3, and VFourier 1 to VFourier 3, meaning for the three coefficients of the harmonic regression of MODIS-derived variables).

**Additional file 2.** The script used for the best modelling algorithms. The script must to be used with the Additional file 3 (see below) and using the freely available software “Orange” (available at https://orange.biolab.si). The script is open to modify any starting conditions of the algorithms and for examining the predictive power of each algorithm for the different species of *Borrelia*.

**Additional file 3.** The dataset with grid data and prevalence categories for the three most commonly reported species of *Borrelia* spp. in the target territory. The dataset must to be used to start the modelling analysis using the script in Additional file 2. It contains data about habitat suitability of the tick vector (column Prob_ricinus) as well as the six temperature (TFourier1 to TFourier 3) and vegetation variables (VFourier1 to VFourier3). Also included (but not used by modelling algorithms in the resolution of the best modelling approach) are “Level 4” (the top category in the hierarchy of bioclimate regions in the LANMPA2 scheme), “GlobCovMaj” (dominant vegetation according to the GlobalCov scheme), “GlobCovDiv” (landscape fragmentation in the cell as the number of different categories of vegetation). The remaining three columns “clas_afzelii”, “clas_garinii” and “clas-valaisiana” refer to the category of repavlence of each of the three modelled species of Bb.

## Abbreviations

Bb: *Borrelia burgdorferi* sensu lato
NDVI: Normalized Difference Vegetation Index
SVM: support vector machine
